# Active case-finding method improves completeness and accuracy of data reported to the rural Eastern Cape Cancer Registry in South Africa

**DOI:** 10.3332/ecancer.2021.1251

**Published:** 2021-06-17

**Authors:** Nontuthuzelo IM Somdyala, Linda Mbuthini, Borna Müller, Nomfuneko Sithole, Akhona Ncinitwa, Debbie Bradshaw

**Affiliations:** 1 South African Medical Research Council, Burden of Disease Research Unit, PO Box 19070, Tygerberg 7505, Cape Town, South Africa; 2 Centre for Lung Infection and Immunity, Department of Medicine, UCT Lung Institute, University of Cape Town, Observatory 7925, Cape Town, South Africa; 3 F. Hoffmann-La Roche Ltd, Pharmaceuticals Division, Global Access, Grenzacherstrasse 124, CH-4070 Basel, Switzerland; 4 Department of Family Medicine and Public Health, University of Cape Town, Observatory 7925, Cape Town, South Africa

**Keywords:** population-based cancer registration, completeness and accuracy, active case finding, resource-distribution disparities, better investment, Africa

## Abstract

The quality and accuracy of the data provided by cancer registries has a significant impact on decision making. Over decades, high-income countries have been successful in monitoring their cancer burden because of well-established data abstraction techniques such as digital systems. Conversely, in low- and middle-income countries, sparsely distributed cancer registries, using alternative less costly, but imprecise methods are struggling to capture all cancer cases. A population-based cancer registry in South Africa covering a resource-limited rural population is faced with challenges in case finding yet the quality and accuracy of the data provided has a significant impact on decision making. The objective of this study was to assess data quality using two data quality attributes ‘completeness and accuracy’ and also to determine the benefits of using active and passive case-finding methods for cancer registration in this population. Data used were collected between January 2014 and December 2015 from four hospitals to compare the quality of both active and passive case-finding methods. From all four hospitals during the same period, a first set of data obtained through passive reporting was compared with a second set of data obtained through active case finding. Covering multiple facilities during active case finding can significantly improve quality of data, while passive case finding is challenged by data collection being confined to one specific health facility, only. Better investment in active case finding is recommended in settings with resource-distribution disparities.

## Introduction

Cancer registries play an important role in monitoring the globally increasing cancer burden. Also, they are of pivotal importance in the planning and evaluation of cancer intervention programmes [1]. Consequently, the quality and accuracy of the data provided by cancer registries has a significant impact on decision making. The International Agency for Research on Cancer (IARC) of the World Health Organization; the global cancer surveillance controlling body, sets the rules and guidelines for cancer registries. Each registry’s data quality is measured against those rules and its value depends on the quality of data generated. In cancer registration, quality of data is described by four attributes: namely, comparability, accuracy/validity, completeness and timeliness [2–6]. However, with the latter, there are no international guidelines at present, although specific standards for abstraction and reporting have been set out by certain organisations [4].

Over decades, high-income countries have been successful in monitoring their cancer burden because of well-established data abstraction techniques such as digital systems [7]. Conversely, in low- and middle-income countries, sparsely distributed cancer registries, using alternative less costly, but imprecise methods are struggling to capture all cancer cases. In Africa, particularly in rural areas, cancer registries are faced with challenges in case finding which include limited resources such as laboratories and oncologist specialists for accurate diagnoses, inaccessibility of hospitals due to poor infrastructure and poor record keeping for tracing and follow-up to patients [8]. This impacts on the generation of reliable, valid and complete data.

The Eastern Cape Cancer Registry (ECCR) is the only rural population-based cancer registry in South Africa covering eight magisterial areas [9–10] with a population of 1.2 million [11]. However, merely collecting cases and establishing a cancer registry is insufficient. Cancer registries have a vested interest in promoting the use of their data and must ensure data generated are of good quality [12]. The ECCR uses both active and passive case-finding methods to mitigate the discrepancies and shortfalls of either method alone [13]. The use of both methods remains financially burdensome to the registry but beneficial in terms of data quality. The active case-finding method involves annual visits to collaborating hospitals by ECCR staff to review and extract all available information from patients’ records with a cancer diagnosis [13]. While the passive case-finding method involves data collectors in only four collaborating hospitals who collect and send data to the ECCR on a monthly basis. For uniformity in both methods, a standardised data collection tool is used [14, 15]. The ECCR contributes to cancer incidence (CI) data globally and regionally; Cancer Incidence in Five Continents (CI5); volumes X and XI [16, 17], Cancer in sub-Saharan Africa [18] and survival collaborative studies; CONCORD 2 and 3 [19, 20]. Other than the achievement of these developmental milestones, no internal study has been conducted to evaluate ECCR data quality. The objective of this study was to assess the quality of data generated by ECCR in two data quality attributes ‘completeness and accuracy’. Completeness is the extent to which all incident cancers occurring in a population are included in the registry [2], whereas accuracy is defined as the proportion of cases in the data with a given characteristics (e.g. topography and morphology) that truly had the attribute [4]. This study focused on these two quality attributes as an initial attempt to checking internal consistency and uniformity of registry records produced using active and passive data collection methods in a resource-limited setting.

## Materials and methods

### Data collection and study sample

This study was conducted between January 2014 and December 2015. In total, 15 hospitals and their associated pathology laboratories constitute the main data source used by the ECCR. Data from these hospitals generally is obtained by registry staff periodically performing patient chart reviews, also termed active case finding. Four of the 15 hospitals are resourced to proactively perform independent data abstraction and reporting to the cancer registry by hospital staff, also termed passive case finding. Data from four hospitals were used to compare the quality of both, the active and passive case-finding methods. From all four hospitals, in 2014 and 2015, a first set of data was obtained through passive reporting. The second dataset was constituted by cases collected by cancer registry staff from the same hospitals during the same period. The four hospitals included in the study were two peripheral hospitals (Tafalofefe and St Elizabeth) and two referral hospitals (Frere and Umtata General Hospital Complex (UGHC)). All types of malignant cancer cases were included and coded according to the International Coding of Disease for Oncology [20].**

### Data and analysis

For this study, the main information used included variables for patients’ identification (name, surname, address, age, sex and ethnicity), the primary organ invaded by the tumour (topography), histological classification of the cancer tissue (morphology), incidence date (first date the patient was seen by the doctor and cancer diagnosed) and healthcare facility in which the patient was first diagnosed with cancer. Variables selected constitute the mandatory variables expected for each case to be accepted as valid in the database. The complete standardised data collection tool used during both active and passive case finding is attached (Appendix 1: Confidential cancer notification form). Before analysis, duplicates were cleaned once the patient’s information has been consolidated.

*(i) Dataset to assess completeness*

For each hospital, completeness of active and passive case finding was assessed by calculating the number of patients captured during 2014 and 2015 by either method divided by the total number of patients identified through both methods combined; new cases active = *N*_a_ and new cases passive = *N*_p_

*(C=N*_a_*T*cases*100 and C=N_p_*T*cases*100). Patients captured by both methods were identical if all identification variables were matching.

*(ii) Dataset to assess accuracy*

**Figure 1.**
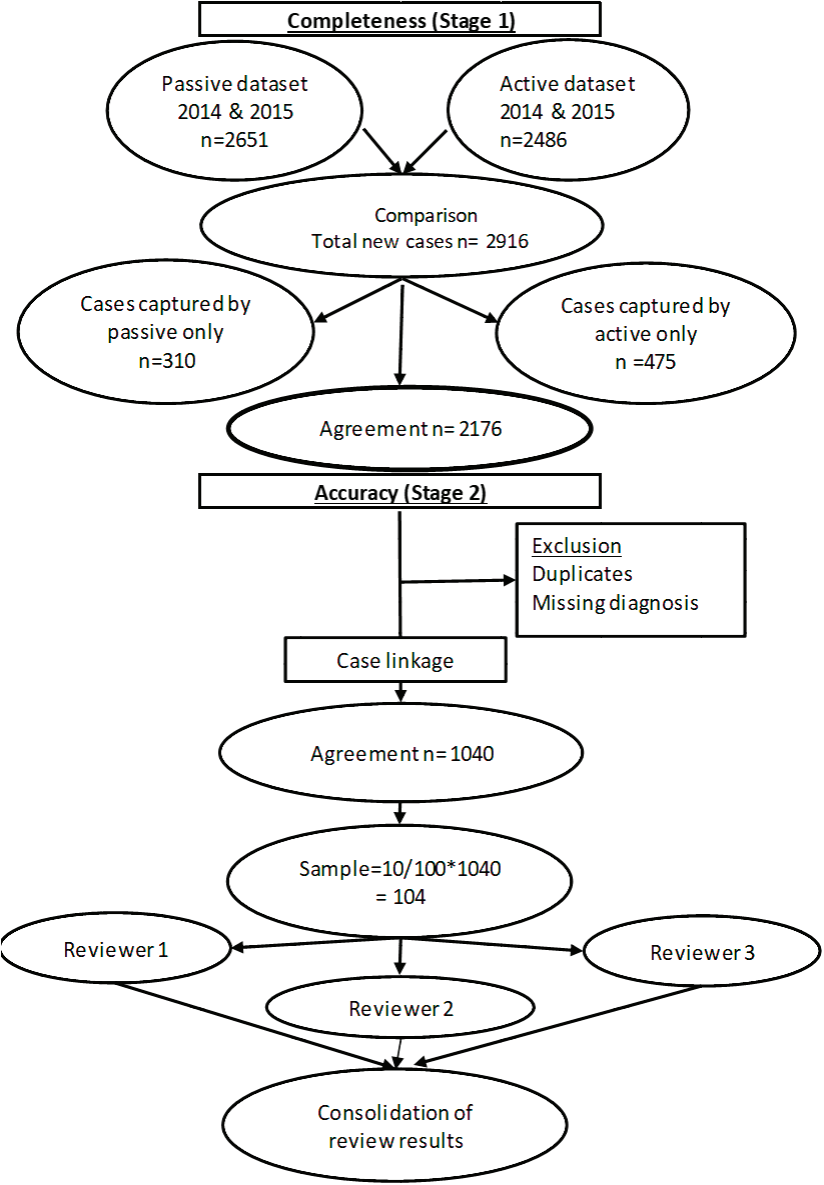
Data evaluation process.

To assess accuracy of information on topography and morphology, data were anonymised by assigning each patient a registry number as identification. Only 1,040 cases recorded by both methods (active and passive) were included in the analysis (Figure 1). Of these, 10% (*N* = 104) were randomly selected to assess and calculate the agreement in information provided with regard to topography and morphology received through both case-finding methods (Figure 1). Three reviewers independently assessed these records and if results differed re-examination was carried out. Subsequently, analysis using Microsoft Excel 2016 was done. Testing for statistically significant differences between active and passive case-finding methods and between hospitals was done in R using McNemar’s and Pearson’s Chi-squared tests, respectively (Table 1a and b).

### Ethics approval

ECCR is an ongoing project of the South African Medical Research Council (SAMRC). The main objective is to generate CI in a defined area. Approval was received from the SAMRC Ethics Committee and permission was sought from the Eastern Cape Department of Health Research Committee. For any additional research which deviates from the original, the principal investigator is expected to review the project proposal. However, for this study, no additional ethics approval was needed as this was a secondary data analysis.

## Results

### Completeness

**Figure 2.**
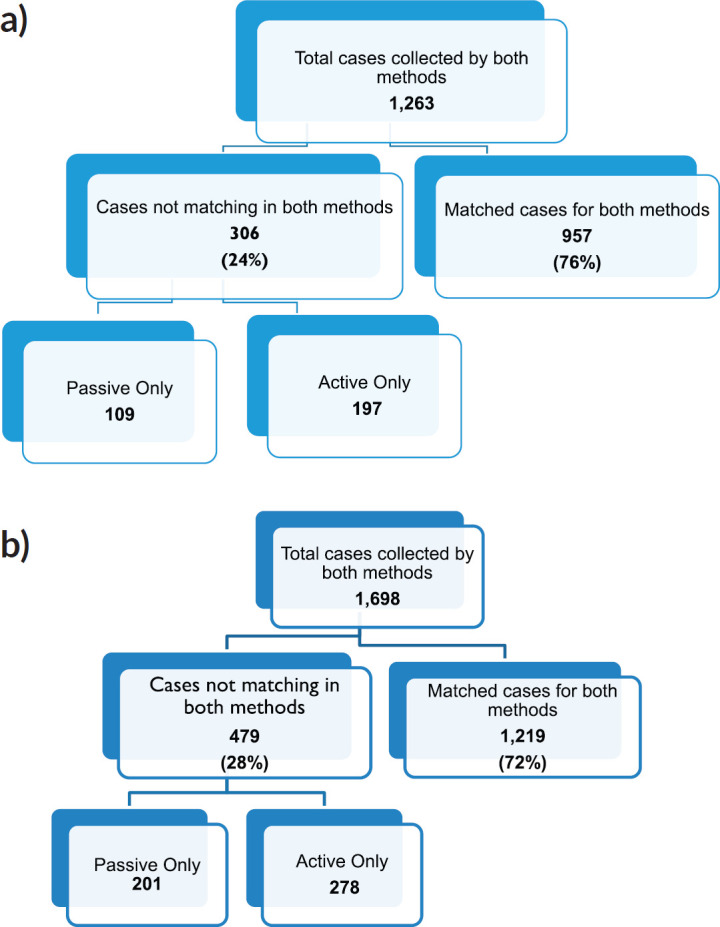
Sorting process for completeness check of case finding in (a): year 2014, (b): year 2015.

We compared cancer registry data obtained through both active and passive case-finding methods from four hospitals in the Eastern Cape Province of South Africa. These consisted of two peripheral hospitals (Tafalofefe and St Elizabeth) and two referral hospitals (Frere and UGHC). During the study period (2014–2015), using active and passive case finding combined, a total of 2,961 individual cancer cases were identified at these four hospitals (Figure 1, Table 2). The number of cases jointly identified by both case-finding methods was 2,176 (74% of the total amount of cases diagnosed; Table 2, Figure 2a and b). Neither of the two methods alone identified all diagnoses reported. However, among all cancer cases diagnosed, active case-finding identified a significantly higher proportion than that from passive case finding (*p* < 0.05; Tables 1a, b and 3).

For the peripheral hospitals St Elizabeth and Tafalofefe, respectively, active case-finding identified 94% and 96% of all cases diagnosed (95% for both peripheral hospitals combined; Table 2). In contrast, passive case finding covered significantly fewer cases among all cases identified (66% and 85%, respectively or 71% for both peripheral hospitals combined; *p* < 0.05; Table 2). Combined for the two peripheral hospitals, adding active case finding to the passive case-finding method increased the number of cancer cases identified by 41% (529 cases were reported through passive case finding alone and 215 cases were added by including active case finding; Tables 2 and 3). In contrast, adding passive case finding to the active case-finding method increased case detection by 6%, only (703 cases were reported through active case finding alone and 41 cases were added by including passive case finding).

The significantly higher performance of active over passive case finding for the peripheral hospitals was not observed for the two referral hospitals Frere and UGHC. For these hospitals combined, among all cancer cases diagnosed, the proportion of cases captured by active or passive case finding alone was very similar (88%; Table 2). Also, adding active case finding to the passive case-finding method still increased the number of cancer cases identified by 13% (Tables 2 and 3).

### Accuracy

**Figure 3.**
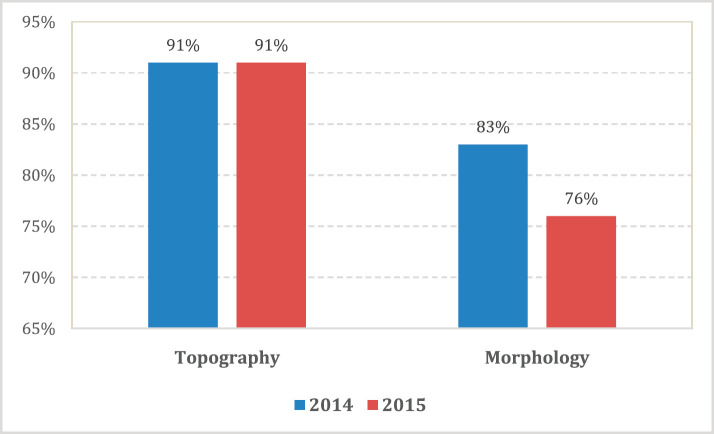
Accuracy; proportion of patient records matched for topography and morphology for both active and passive data case-finding methods by year.

An assessment of the accuracy of information abstracted from the medical records by each method was done with regard to data on tumour topography and morphology. The overall proportion of patient records matching for tumour topography was 89% in 2014 and 90% in 2015 (Table 4). However, agreement for morphology was less with 83% in 2014 and 76% in 2015 (79% for both years combined; Table 5, Figure 3).

Specifically, for data on tumour topography, at both peripheral hospitals, agreement of active and passive case-finding methods was 100%; however, at a very low sample size (*N* = 19 records in total). For referral hospitals, agreement was 87% at UGHC and 92% at Frere (89% in total; *N* = 85; Table 4).

For data on tumour morphology, at peripheral hospitals, agreement was 82% at St Elizabeth and 75% at Tafalofefe (79% in total; *N* = 19; Table 5). For referral hospitals, agreement of active and passive case finding was 71% at UGHC and 85% at Frere (79% in total; *N* = 85; Table 5).****

## Discussion

The main objective of this study was to assess the completeness and accuracy of cancer patients’ data reported to a rural population-based cancer registry in South Africa. Data on the burden of cancer in Sub-Saharan Africa are very scarce and the limited information available is mostly biased towards urban centres with better infrastructure and possibly distinct disease epidemiology. Our study contributes significantly to the understanding of the importance of CI even in rural populations of Africa with resource limitations.

**Figure 4.**
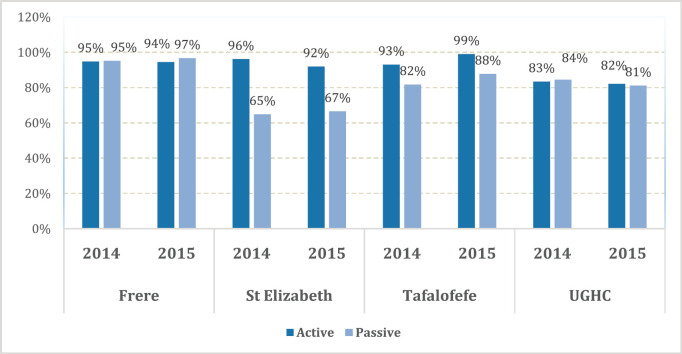
Completeness; proportion of cancer cases reported by both active and passive case-finding methods by facility.

The overall results of the study indicated less variations with regard to completeness compared to accuracy; with a range of 83%–96% in referral hospitals and 83% in peripheral hospitals (Figure 4). However, differences were noted in individual hospitals with a range of 65%–97%. Neither of the two methods alone identified all patients reported. Since registry staff reviewed the same records used by data collectors during data abstraction, human error in recording resulted in missing cases in either case-finding method. However, in the entire study, active case-finding contributed more cases than passive (*p* < 0.05). We also observed that for the resource-constrained peripheral hospitals, active case-finding captured a significantly larger portion of the total number of patients recorded than passive case finding (95% versus 71%). Hence, active case finding significantly improved case detection; increasing the number of cases identified by as much as 41%. At better equipped referral hospitals, passive and active case finding identified a similar proportion of patients among all cases recorded (88%). Nonetheless, also for referral hospitals, active case finding significantly increased the number of patients captured by 13%.

A higher accuracy agreement for data on topography was observed with disagreement in only 17%–21% of records which is not a nugatory result especially in under-resourced settings where there is manual data collection. Passive case-finding led to poor information recorded on cancer morphology compared to active. Disagreement was frequently based on different or incorrect information recorded regarding diagnoses. A higher record of accuracy was observed in referral compared to peripheral hospitals. Frere Hospital, which is a referral, showed the highest accuracy in recording. This hospital unlike the others has an in-house pathology laboratory linked data to patients’ records. The hospital is also fully equipped with oncology and radiation units and several practicing oncologists and registrars. Similar performance of passive reporting (Appendix 2: Resource differences between hospitals observed during the study, possibly impacting quality of passive reporting) was reported at UGHC Hospital which is a referral hospital and at Tafalofefe Hospital which although is a peripheral hospital has a similar infrastructure which is supportive and relevant to cancer registration. In contrast, St Elizabeth Hospital had the lowest completeness for passive case-finding (Table 2) due to frequently interrupted data submissions, incorrect record of information regarding diagnosis which raised some concern. Information regarding high morphology disagreement reflects the absence of oncology trained specialists at both peripheral hospitals and lack of follow-up of referred cases so that information about diagnosis is completed and improved.

This is the first study of its kind in South Africa and one of very few in Africa due to the general scarcity of cancer registries in this continent. The results highlight the challenges experienced in achieving cancer registration with stable good quality data. These include lack of funding to support these registries, political will and commitment to support data generation activities. Consequently, there is no infrastructure (geographical remoteness, no transport, etc.), human (oncologists) and material resources (laboratories, proper documentation, etc.) and inaccessibility of health medical care centres.

Similar studies include one by Al-Haddad *et. al*. [8] whose findings in Nigeria are similar to our study, because both found evidence of incompleteness in case finding. This was attributed to infrastructure challenges including human and material resources for proper diagnosis and data recording. There is urban bias observed in another study conducted in Tanzania; of the Kilimanjaro Cancer Registry [22]. High performance was evident with 98% completeness and 94% accuracy and can be linked with the number of specialists in the hospital which tends to improve the quality of data collection [4]. The same was observed in two recent studies from the high-income countries: Singapore (Fung *et. al* [23]) and Switzerland (Wanner *et. al* [24]). In both studies, infrastructure is not an issue, but these registries showcase the importance of high-quality data which is important for confidence in published results and receiving trust of those who use data including public health planners.

### Limitations

Our study has some limitations. The study population was small; only two peripheral and two referral hospitals were included, which may only be marginally representative of the reporting capabilities of other hospitals in South Africa. Furthermore, their results cannot be compared within the ECCR as they are the only hospitals using the passive case-finding method which constitute only 20% of the total case-finding methods. Patients were double counted especially those collected by active case finding. However, use of multiple sources improves the quality of diagnoses. These limitations did not impact on the general findings of our study but highlighted the challenges of passive reporting especially in resource-limited areas.

## Conclusion

Distinct hospital-specific challenges in cancer registration were observed, particularly in peripheral hospitals. More than a third of the patients diagnosed would not have been reported. Infrequent reporting is associated with staff shortage resulting in delays in reporting and missing information in some cases. This study highlights the importance of regular training for data collectors and retain them in the project for longer time exposure and experience to improve their understanding of concepts such as ‘completeness and accuracy’ in cancer registration and impact of these in cancer epidemiology. Furthermore, the passive case-finding method alone has negative implications for the quality of data. Two methods used complement each other particularly in settings like South Africa with disparities in resource distribution. It was also evident that utilising and access to multiple sources including pathology laboratories during active case finding improved data quality in the ECCR.

Cancer registration in Africa is possible but it has proved to be a difficult task due to many factors, some of which include: difficulty in tracing cases, lack of medical information systems and lack of funding or underfunding. Africa must seriously consider investing in special staff training for efficient digital information exchange between external pathology laboratories and hospitals to address these challenges. Investment in active case finding will be cost effective and is recommended.

## Figures and Tables

**Table 1.** (a) How did the performance of active and passive case finding differ between peripheral and referral hospitals?

By year, *p*-values for pairwise comparison of the sensitivity of active and passive case finding between peripheral and referral hospitals using Pearson's Chi-squared test with Yates' continuity correction		
**Year**	**Active**	**Passive**
2014	0.54	**0.01**
2015	0.37	**0.04**
Both years	0.25	**0.00**

*p*-values reaching statistical significance are highlighted in bold

**Table 1.** (b) How did the performance of active case finding differ between hospitals?

*p*-values for pairwise comparison of the sensitivity of active case finding between hospitals (all years combined) using Pearson's Chi-squared test with Yates' continuity correction				
	**St Elizabeth**	**Tafalofefe**	**UGHC**	**Frere**
St Elizabeth		0.92	0.09	0.96
Tafalofefe			0.18	0.95
UGHC				**0.03**
Frere				
*p*-values reaching statistical significance are highlighted in bold				

**How did the performance of passive case finding differ between hospitals?**				
*p*-values for pairwise comparison of the sensitivity of passive case finding between hospitals (all years combined) using Pearson's Chi-squared test with Yates' continuity correction				
	**St Elizabeth**	**Tafalofefe**	**UGHC**	**Frere**
St Elizabeth		**0.05**	**0.01**	**0.00**
Tafalofefe			0.84	0.28
UGHC				**0.01**
Frere				

*p*-values reaching statistical significance are highlighted in bold

**Table 2.** Cancer cases registered by detection method, hospital and year

Case detection method	St Elizabeth*		Tafalofefe*		UGHC**		Frere**		Peripheral		Referral		Grand Total	
	Freq	Se	Freq	Se	Freq	Se	Freq	Se	Freq	Se	Freq	Se	Freq	Se
*2014*														
Active	249	96.1%	107	93.0%	327	83.4%	471	94.8%	356	95.2%	798	89.8%	1154	91.4%
**Passive**	168	64.9%	94	81.7%	331	84.4%	473	95.2%	262	70.1%	804	90.4%	1066	84.4%
**Active not passive**	91	35.1%	21	18.3%	61	15.6%	24	4.8%	112	29.9%	85	9.6%	197	15.6%
**Passive not active**	10	3.9%	8	7.0%	65	16.6%	26	5.2%	18	4.8%	91	10.2%	109	8.6%
**Active and passive**	158	61.0%	86	74.8%	266	67.9%	447	89.9%	244	65.2%	713	80.2%	957	75.8%
**Active or passive (all cases)**	**259**	**100.0%**	**115**	**100.0%**	**392**	**100.0%**	**497**	**100.0%**	374	**100.0%**	889	**100.0%**	**1263**	**100.0%**
*2015*														
**Active**	250	91.9%	97	99.0%	691	82.1%	459	94.4%	347	93.8%	1150	86.6%	1497	88.2%
**Passive**	181	66.5%	86	87.8%	683	81.1%	470	96.7%	267	72.2%	1153	86.8%	1420	83.6%
**Active not passive**	91	33.5%	12	12.2%	159	18.9%	16	3.3%	103	27.8%	175	13.2%	278	16.4%
**Passive not active**	22	8.1%	1	1.0%	151	17.9%	27	5.6%	23	6.2%	178	13.4%	201	11.8%
**Active and passive**	159	58.5%	85	86.7%	532	63.2%	443	91.2%	244	65.9%	975	73.4%	1219	71.8%
**Active or passive (all cases)**	**272**	**100.0%**	**98**	**100.0%**	**842**	**100.0%**	**486**	**100.0%**	370	**100.0%**	1328	**100.0%**	**1698**	**100.0%**
*Both years combined*														
**Active**	499	94.0%	204	95.8%	1018	82.5%	930	94.6%	703	94.5%	1948	87.9%	2651	89.5%
**Passive**	349	65.7%	180	84.5%	1014	82.2%	943	95.9%	529	71.1%	1957	88.3%	2486	84.0%
**Active not passive**	182	34.3%	33	15.5%	220	17.8%	40	4.1%	215	28.9%	260	11.7%	475	16.0%
**Passive not active**	32	6.0%	9	4.2%	216	17.5%	53	5.4%	41	5.5%	269	12.1%	310	10.5%
**Active and passive**	317	59.7%	171	80.3%	798	64.7%	890	90.5%	488	65.6%	1688	76.1%	2176	73.5%
**Active or passive (all cases)**	**531**	**100.0%**	**213**	**100.0%**	**1234**	**100.0%**	**983**	**100.0%**	744	**100.0%**	2217	**100.0%**	**2961**	**100.0%**

* Peripheral hospitals

** Referral hospitals

Freq: Absolute number of cases detected

Se: Sensitivity of case detection method for hospital and period indicated

Active: Cases registered by applying active case finding methods

Passive: Cases registered by applying passive case finding methods

**Table 3.** Improvement of case registration by active case finding method

Year	St Elizabeth*	Tafalofefe*	UGHC**	Frere**	Peripheral	Referral	All
2014	54.2%	22.3%	18.4%	5.1%	42.7%	10.6%	18.5%
2015	50.3%	14.0%	23.3%	3.4%	38.6%	15.2%	19.6%
Both years combined	52.1%	18.3%	21.7%	4.2%	40.6%	13.3%	19.1%

The proportions indicate the cases added by active case finding relative to the number reported by passive case finding

* Peripheral hospitals

** Referral hospitals

**Table 4.** Agreement of topographic data between active and passive case finding.

Case detection method	St Elizabeth*		Tafalofefe*		UGHC**		Frere**		Peripheral		Referral		Grand Total	
	Freq	%	Freq	%	Freq	%	Freq	%	Freq	%	Freq	%	Freq	%
*2014*														
**Agreement**	5	100.0%	4	100.0%	10	83.3%	23	92.0%	9	100.0%	33	89.2%	42	91.3%
**Disagreement**	0	0.0%	0	0.0%	2	16.7%	2	8.0%	0	0.0%	4	10.8%	4	8.7%
**Total**	**5**	**0.0%**	**4**	**0.0%**	**12**	**16.7%**	**25**	**8.0%**	9	**0.0%**	37	**10.8%**	**46**	**8.7%**
*2015*														
**Agreement**	6	100.0%	4	100.0%	23	88.5%	20	90.9%	10	100.0%	43	89.6%	53	91.4%
**Disagreement**	0	0.0%	0	0.0%	3	11.5%	2	9.1%	0	0.0%	5	10.4%	5	8.6%
**Total**	**6**	**0.0%**	**4**	**0.0%**	**26**	**11.5%**	**22**	**9.1%**	10	**0.0%**	48	**10.4%**	**58**	**8.6%**
*Both years combined*														
**Agreement**	11	100.0%	8	100.0%	33	86.8%	43	91.5%	19	100.0%	76	89.4%	95	91.3%
**Disagreement**	0	0.0%	0	0.0%	5	13.2%	4	8.5%	0	0.0%	9	10.6%	9	8.7%
**Total**	**11**	**100.0%**	**8**	**100.0%**	**38**	**100.0%**	**47**	**100.0%**	19	**100.0%**	85	**100.0%**	**104**	**100.0%**

* Peripheral hospitals

** Referral hospitals

Freq: Absolute number of cases

%: Percentage relative to total for the year and hospital indicated

active/passive: Information registered by applying active/passive case finding methods

**Table 5.** Agreement of morphological data between active and passive case finding.

Case detection method	St Elizabeth*		Tafalofefe*		UGHC**		Frere**		Peripheral		Referral		Grand Total	
	Freq	%	Freq	%	Freq	%	Freq	%	Freq	%	Freq	%	Freq	%
*2014*														
**Agreement**	4	80.0%	2	50.0%	10	83.3%	22	88.0%	6	66.7%	32	86.5%	38	82.6%
**Disagreement**	1	20.0%	2	50.0%	2	16.7%	3	12.0%	3	33.3%	5	13.5%	8	17.4%
**Total**	**5**	**20.0%**	**4**	**50.0%**	**12**	**16.7%**	**25**	**12.0%**	9	**33.3%**	37	**13.5%**	**46**	**17.4%**
*2015*														
**Agreement**	5	83.3%	4	100.0%	17	65.4%	18	81.8%	9	90.0%	35	72.9%	44	75.9%
**Disagreement**	1	16.7%	0	0.0%	9	34.6%	4	18.2%	1	10.0%	13	27.1%	14	24.1%
**Total**	**6**	**16.7%**	**4**	**0.0%**	**26**	**34.6%**	**22**	**18.2%**	10	**10.0%**	48	**27.1%**	**58**	**24.1%**
*Both years combined*														
**Agreement**	9	81.8%	6	75.0%	27	71.1%	40	85.1%	15	78.9%	67	78.8%	82	78.8%
**Disagreement**	2	18.2%	2	25.0%	11	28.9%	7	14.9%	4	21.1%	18	21.2%	22	21.2%
**Total**	**11**	**100.0%**	**8**	**100.0%**	**38**	**100.0%**	**47**	**100.0%**	19	**100.0%**	85	**100.0%**	**104**	**100.0%**

* Peripheral hospitals

** Referral hospitals

Freq: Absolute number of cases

%: Percentage relative to total for the year and hospital indicated

active/passive: Information registered by applying active/passive case finding methods

## Appendixes

### Appendix 2. Resource differences between hospitals observed during the study, possibly impacting quality of passive reporting.

**Table:**

Parameter	St Elizabeth*	Tafalofefe*	UGHC**	Frere**
Completeness of passive reporting***	65.7%	84.5%	82.2%	95.9%
Pathology laboratory data linked to hospital patient data	✗	✗	✗	✓
Oncologists present during study period	✗	✗	✗	✓
Oncology-trained nurses present during study period	✓	✓	✓	✓
Uninterrupted reporting of new cancer cases	✗	✓	✓	✓

* Peripheral hospitals

** Referral hospitals

*** Proportion of cases reported by passive case finding among all patients captured over both study years combined

Freq: Absolute number of cases

%: Percentage relative to total for the year and hospital indicated

active/passive: Information registered by applying active/passive case finding methods
